# University of Pennsylvania, Department of Veterinary Medicine

**Published:** 1900-01

**Authors:** Leonard Pearson

**Affiliations:** Office of the Dean, Philadelphia


### COMMUNICATIONS.

### UNIVERSITY OF PENNSYLVANIA, DEPARTMENT OF VETERINARY MEDICINE.

OFFICE OF THE DEAN, PHILADELPHIA, January 17, 1900.

DEAR SIR: It has for many years been the practice of the leading physicians in nearly every community to go from time to time to one of the centres of medical education for the purpose of pursuing post-graduate courses of instruction. In this way they have been enabled to keep in touch with the advances in medical science and to freshen their knowledge. Veterinarians, on the other hand, have not adopted this practice, although the need for it cannot be less among members of our profession than among physicians. This condition is, no doubt, due in part to the fact that no arrangements have been made for providing short courses in the kind of work that practitioners of veterinary medicine wish to pursue.

It is now proposed to offer a six-day practical course in State veterinary medicine, bacteriology, meat-inspection, and dairy inspection. The plan provides also for clinical demonstrations in surgery, for visits to at least two of the best dairy farms in the country, and for free discussion by the members of the class of all of the subjects taken up in the course.

This course is provided especially for the veterinarians of Pennsylvania who are interested in the broader public aspects of veterinary science (those who act as inspectors for the State Livestock Sanitary Board), but it will include instruction in many subjects of use in everyday private practice.

Among those who will take part in the discussions are Drs.

Ravenel, Harger, Adams, Hoskins, Conard, Klein, and Pearson.

It is proposed to begin this course at the Veterinary Department of the University of Pennsylvania, Tuesday, February 27, 1900, at 8.30 A.M. This time has been selected because the State Veterinary Society meets on Tuesday, March 6th, and it is known that many veterinarians will come from distant parts of the State to attend this meeting. By coming one week earlier, advantage can be taken of the special course and the meeting at one visit.

While there is always some difficulty in leaving a practice for a week or ten days, it is to be remembered that the purpose in so doing is to acquire knowledge and experience that will make the services of the veterinarian of more value to his clients.

There will be no charge for tuition and no other fee, so the only expense will be for board and railroad fare.

This course is offered to all legally registered veterinarians in Pennsylvania.

Those who wish to take this course are requested to send their names as soon as possible to the undersigned.

Respectfully yours,
LEONARD PEARSON.

